# Beyond Crossing Fibers: Bootstrap Probabilistic Tractography Using Complex Subvoxel Fiber Geometries

**DOI:** 10.3389/fneur.2014.00216

**Published:** 2014-10-28

**Authors:** Jennifer S. W. Campbell, Parya MomayyezSiahkal, Peter Savadjiev, Ilana R. Leppert, Kaleem Siddiqi, G. Bruce Pike

**Affiliations:** ^1^McConnell Brain Imaging Centre, Montreal Neurological Institute, McGill University, Montreal, QC, Canada; ^2^Psychiatry Neuroimaging Laboratory, Brigham and Women’s Hospital, Harvard Medical School, Boston, MA, USA; ^3^Laboratory for Mathematics in Imaging, Brigham and Women’s Hospital, Harvard Medical School, Boston, MA, USA; ^4^Centre for Intelligent Machines, McGill University, Montreal, QC, Canada; ^5^Department of Radiology, Hotchkiss Brain Institute, University of Calgary, Calgary, AB, Canada

**Keywords:** diffusion MRI, fiber orientation distribution function, high angular resolution diffusion imaging, fiber dispersion, curve inference

## Abstract

Diffusion magnetic resonance imaging fiber tractography is a powerful tool for investigating human white matter connectivity *in vivo*. However, it is prone to false positive and false negative results, making interpretation of the tractography result difficult. Optimal tractography must begin with an accurate description of the subvoxel white matter fiber structure, includes quantification of the uncertainty in the fiber directions obtained, and quantifies the confidence in each reconstructed fiber tract. This paper presents a novel and comprehensive pipeline for fiber tractography that meets the above requirements. The subvoxel fiber geometry is described in detail using a technique that allows not only for straight crossing fibers but for fibers that curve and splay. This technique is repeatedly performed within a residual bootstrap statistical process in order to efficiently quantify the uncertainty in the subvoxel geometries obtained. A robust connectivity index is defined to quantify the confidence in the reconstructed connections. The tractography pipeline is demonstrated in the human brain.

## 1 Introduction

This paper describes a pipeline for performing fiber tractography using a complex description of the subvoxel fiber geometry within a bootstrap probabilistic framework. A *curve inference* algorithm that can accurately describe the fiber geometry of straight, crossing, bending, and fanning fibers on a subvoxel scale is examined. Given this probabilistic description of the subvoxel fiber geometry, confidences are assigned to individual fiber tract segments and subsequently to entire reconstructed tracts, using a *weakest link* connectivity measure. These advancements demonstrate promise for accurately mapping the white matter fiber bundles of the healthy human brain, and may provide improved sensitivity for tracking fiber changes in disease processes.

Diffusion magnetic resonance imaging (MRI) is the first non-invasive method capable of exploring neural connectivity and reconstructing white matter fiber structure *in vivo*. Diffusion MRI is able to probe white matter fiber orientation because water diffusion is anisotropic in brain, with greater displacement of water molecules parallel to white matter fiber tracts. This characteristic can be used to reconstruct connectivity patterns between different cortical/subcortical areas of the brain. The first step in diffusion MRI tractography is estimation of the diffusion probability density function (PDF) describing the anisotropic diffusion of water molecules. Several techniques have been developed to compute the diffusion PDF, ranging from diffusion tensor imaging (DTI) (2), which is a low angular resolution technique, to high angular resolution diffusion imaging (HARDI) techniques, such as diffusion spectrum imaging (DSI) (30) and q-ball imaging (QBI) (31). While DTI was the first successful technique in modeling the diffusion PDF, it fails to extract the true fiber structure within a voxel containing a crossing, branching, or merging configuration of fibers due to its underlying assumption of a single anisotropic Gaussian PDF. Using the latter techniques, a high angular resolution diffusion orientation distribution function (ODF) can be obtained, which has the potential to model multiple fiber orientations within a voxel. More recent techniques have been developed for calculation of the fiber ODF (1, 8, 26, 27), which is the diffusion ODF deconvolved with a single fiber response function. The fiber ODF can be calculated directly by deconvolution of the MRI signal profile with the single fiber response function in signal space [e.g., Ref. (27)], or by calculating a diffusion ODF with q-space techniques and deconvolving with the single fiber response function in diffusion ODF space [e.g., Ref. (8)].

Regardless of the method used for the computation of diffusion ODFs or fiber ODFs, there are always uncertainties associated with the estimated fiber orientations. These uncertainties, which can be due either to acquisition noise or model deficiencies, should be incorporated in further processing, such as tractography, in order to reflect the confidence in the reconstructed fiber pathways. To address this issue, there has been a large body of research dedicated to probabilistic tractography. These probabilistic methods can be divided into two groups: those that model noise parameters by some probability distribution (3, 21) and bootstrap based methods that capture the uncertainty in the data by random selection from a set of different measurements (15). The bootstrap is a statistical technique that allows estimation of a given distribution using data resampling (9). While traditional bootstrap methods can provide a non-parametric estimation of diffusion uncertainty, the need for multiple data acquisitions hinders any practical application of them to HARDI based techniques. A more viable alternative to the standard bootstrap method is the residual bootstrap, which requires only a single HARDI measurement. It has recently been proposed in the context of q-ball imaging (5, 12), and has subsequently been applied to spherical deconvolution diffusion MRI reconstruction (13, 14, 18).

Fiber ODFs obtained from HARDI measurements, combined with a bootstrap probabilistic approach, can characterize uncertainties in the fiber orientation well in the case of straight fiber structure. However, despite the significant improvement that can be expected using this technique, there still exist ambiguities in the subvoxel fiber structure that cannot be resolved by fiber ODFs. An example is the failure to discriminate between a fanning and curving fiber tract. The fiber ODF for these two geometries is identical, depicting a continuous range of smoothly varying fiber directions. Fiber orientation dispersion has been characterized by several models (25, 32), and curving and fanning fibers exist throughout the brain at a wide range of length scales. In recent work by Savadjiev et al. (24), the issue of differentiating curving from fanning was addressed by implementing a 3D *curve inference* algorithm that assigns different labels to such ambiguous configurations. Improvement was shown in tractography using the curve inference labeling information, which differentiates single, fanning and crossing fiber configurations, and gives the polarity of the fanning in the case of fanning.

By properly incorporating uncertainty due to noise in tractography, false negative results can be reduced. This is of particular importance for tractography applications such as surgical planning. By incorporating accurate descriptions of the subvoxel fiber geometry, false positive and false negative results can be reduced. For example, tracking algorithms that fan wherever the fiber ODF is broad may result in connections that could be eliminated by characterizing the polarity of the fanning and fanning only in one direction. This is of benefit when characterizing anatomy in detail.

In this paper, the curve inference algorithm, which allows for straight, crossing, bending, and diverging fibers on the subvoxel scale, is used as an example technique to accurately describe the fiber geometry; the pipeline can be extended to handle other subvoxel fiber geometries, such as bottlenecks. Diffusion MRI tractography is performed using the output of the bootstrap curve inference process, which allows us to define a confidence value for each reconstructed tract, and subsequently an index of connectivity, at each voxel in the volume, to the region of interest (ROI) of the user’s choice. This connectivity index is derived using a *weakest link* approach (6, 22), and solves many of the problems inherent in popular connectivity indices that are based on *frequency of connection* (4, 17, 21), which count the number of times probabilistic streamlines pass through voxels. Our results demonstrate the promise of this pipeline in the healthy human brain. We also evaluate how well the residual bootstrap predicts scan-rescan repeatability of fiber orientation estimates.

## 2 Materials and Methods

### 2.1 Acquisition

MRI data were acquired for three healthy subjects on a Siemens 3 T Trio MR scanner (Siemens Medical Systems, Erlangen, Germany) using an eight-channel phased-array head coil. The study was approved by the Montreal Neurological Institute Research Ethics Board, and informed consent was obtained prior to the study. Diffusion encoding was achieved using a single-shot spin-echo echo planar sequence with twice-refocused balanced diffusion encoding gradients. A dataset designed for high angular resolution reconstruction was acquired with 99 diffusion encoding directions, 2 mm isotropic voxel size, 63 slices, *b* = 3000 s/mm^2^, TE = 121 ms, TR = 11.1 s, and GRAPPA parallel reconstruction with an acceleration factor of two. A 1 mm isotropic resolution T_1_ weighted anatomical scan was also acquired (TR = 9.7 ms, TE = 4 ms, α = 12°). For one subject, the diffusion-weighted acquisition was repeated four times, without repositioning.

A second diffusion MRI protocol that is more commonly used (16) was also explored for one subject. This protocol provides lower angular resolution, but higher signal-to-noise ratio and a shorter acquisition time. It used a *b*-value of 1000 s/mm^2^ and 64 diffusion encoding directions, 2 mm isotropic voxel size, 65 slices, TE = 92 ms, TR = 9.3 s, and GRAPPA parallel reconstruction with an acceleration factor of two.

### 2.2 Probabilistic deconvolution

All of the data processing was implemented in C++^1^. The diffusion-weighted signal profiles were fit to a spherical harmonic (SH) basis of order eight, using a least-squares estimation. Multiple (in this experiment, 100 repetitions) diffusion-weighted signal profiles were generated using the residual bootstrap method. For each repetition, the residuals from the SH fit of the original diffusion-weighted signal profiles were added at random with replacement to the SH profile to generate a new diffusion-weighted signal profile reflecting the noise characteristics of the acquisition. This residual bootstrap procedure follows the approach of Berman et al. (5), originally applied to q-ball imaging. The residuals *r_i_* were first corrected for leverage using the following factor:

(1) $${\hat{r}}_{i}=\frac{r_{i}}{\sqrt{\left(1-h_{i}\right)}},$$

where *h_i_* are the diagonal elements of the hat matrix H, which is the matrix that maps the original signal values *S* to the SH-fitted signal values Ŝ:

(2) Ŝ=HS.

The new diffusion-weighted signal profile was checked for negative values, but these did not occur using data of the quality typical of human *in vivo* diffusion MRI acquisitions. For the noisier, *b* = 3000 s/mm^2^, data, the corrected residual magnitude on average in the brain was 4.0 ± 3.5% of the signal profile intensity. For each bootstrap repetition, the new signal profile was input to a spherical deconvolution algorithm [an adaptation of the approach of Anderson (1)], using a spherical harmonic expansion of order eight. The pipeline can be run with any spherical deconvolution algorithm, e.g., constrained non-negative spherical deconvolution (27) would be appropriate.

### 2.3 Probabilistic curve inference algorithm: Labeling of subvoxel fiber configurations and construction of PDF for fiber propagation

The deconvolved ODFs were used as input to a curve inference algorithm, the details of which are described by Savadjiev et al. (23, 24). The curve inference process labels the subvoxel fiber configuration for each bootstrap repetition as either fanning, single, or multiple (i.e., crossing) curves, and gives the polarity in the case of fanning. The input to the curve inference algorithm is the fiber ODF obtained from spherical deconvolution. The 3D curve inference algorithm exploits the local differential geometry of 3D curves to infer the likely local curves modeled as helices. A co-helicity measure is defined to compute the degree of compatibility among triplets of orientations. The algorithm then defines what *curves* exist in each voxel, as opposed to what *straight lines* exist, thereby allowing fanning and subvoxel curvature to be distinguished. In this implementation, only voxels with one fiber ODF maximum are considered as possible fannings. Hence, voxels that have a broad fiber ODF will be labeled as fannings if the local neighborhood fibers support this configuration, and otherwise the breadth of the fiber ODF is assumed to be due to curvature. In this paper, *curve inference labeling* refers to this process of identifying voxels as containing fibers in one of several geometries including fanning.

**Figure 1:**
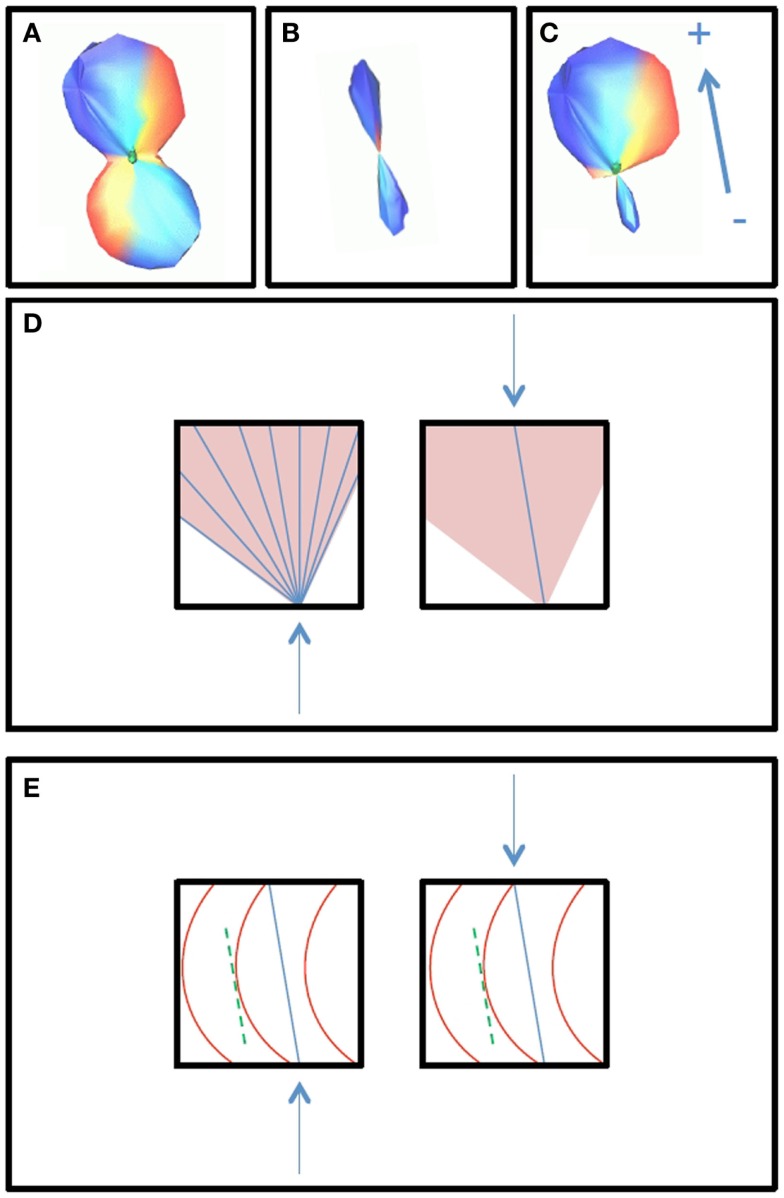
**The construction of the PDF for the direction of tractography is shown**. Broad fiber ODFs, shown in box **(A)**, may be due to either fanning or curvature, and curve inference distinguishes between these cases. In the case of fanning, fanning of the streamlines propagated in tractography will occur in only one direction [+, as indicated by the polarity vector in box **(C)**], while in the other direction, there is a merge [− in box **(C)**]. In the merge direction, and in the case of curvature, tractography follows non-zero directions in the PDF for the fiber ODF maximum [box **(B)**]. The PDF for the direction of propagation for curvature is then equal to the PDF for the fiber ODF maximum **(B)**, and the PDF for the direction of propagation for fanning/merging **(C)** is the combination of the average fiber ODF **(A)** and the PDF for its maximum **(B)**. Box **(D)** illustrates how the streamlines will propagate using FACT integration in the cases of fanning (left) and merging (right). Box **(E)** illustrates how the streamlines will propagate in the case of curvature. The fiber ODF maximum is assumed to represent the intermediate tangent to the curve, which is used as a discretized approximation to the curve segment in the voxel, entering and exiting the voxel where the curve would have done.

**Figure 2:**
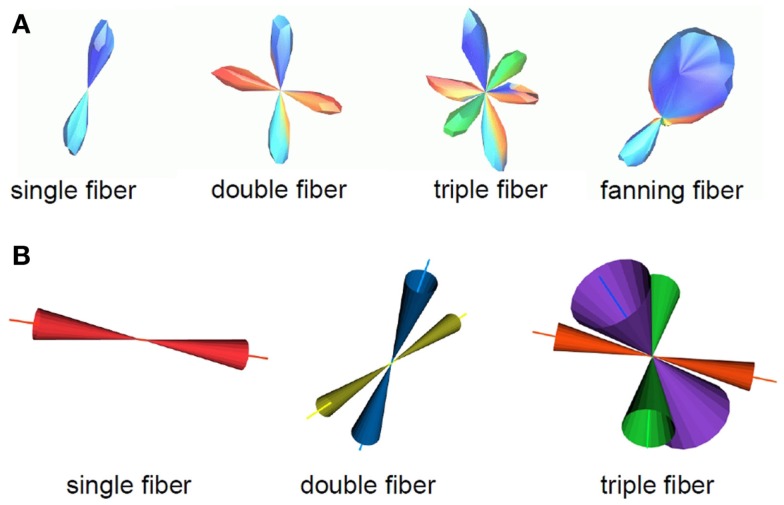
**A summary of the fiber configurations obtained from the probabilistic curve inference labeling is shown**. **(A)** PDFs for the direction of propagation of tractography. The algorithm produces output for four possible configurations, which are, from left to right, a single (potentially curving) fiber bundle, double crossing, triple crossing, and fanning fibers. These distributions come from one *b* = 3000 s/mm^2^ dataset on which curve inference was run. **(B)** Confidence intervals for the mean of these distributions are also obtained. Here, the 95% confidence intervals for the mean fiber ODF maximum are shown for the non-fanning cases, using the *b* = 1000 s/mm^2^ dataset.

The outputs of the curve inference labeling algorithm are the geometry labels assigned to each voxel and a vector describing the fanning fiber polarity. Combined with the fiber ODFs and their maxima, we can construct a PDF for the direction of propagation of tractography. In this implementation, only crossings of up to three fibers are considered, as four way crossings were not detected with the angular and spatial resolution employed in this study. The algorithm can be extended to handle an arbitrary number of fiber ODF maxima. On each bootstrap repetition, a given voxel will be labeled a fanning or a single fiber (if the fiber ODF has one maximum and it is not a fanning), a double crossing (if the fiber ODF has two maxima), or a triple crossing (if the fiber ODF has three maxima). If it is a fanning, the full fiber ODF is saved. The broad extent of the fiber ODF is assumed to represent the range of fiber orientations in the fan. In all cases, the fiber ODF maxima are saved. These will be the directions of propagation for the merge direction of a fan, for the single (potentially curving) fiber, for the double fiber crossing (if there are two maxima), and for the triple fiber crossing. From the bootstrap repetitions, we then have an average fiber ODF (Figure 1A), and an average ODF maximum (or maxima) (Figure 1B). The PDF for the direction of propagation of tractography for fanning is the combination of the two: the mean fiber ODF in the direction of the fan polarity vector, and the mean fiber ODF maximum in the direction of the merge, which is opposite to the fan polarity vector (Figure 1C). Figure 2A summarizes the possible PDFs for the direction of propagation of tractography.

The mean vectors and bootstrap confidence intervals for the fiber directions were also computed. In the case of crossing, the distinct crossing fibers were treated separately. Mean vectors and cones of uncertainty for the 95% confidence intervals for the mean vector are shown in Figure 2B, using the *b* = 1000 s/mm^2^ dataset as an example. The PDF for the direction of propagation for each fiber was normalized to have volume one. Occurrence rates were computed for each of the four fiber geometries at each voxel, equal to the number of times a specific geometry label has been assigned to that voxel. As a final step, the vectors obtained for each different configuration were matched to obtain an occurrence rate, *O_v_*, of each *vector*. Specifically, if a vector is labeled as a single fiber direction on some repetitions, and matches one of many crossing fiber directions on other repetitions, and falls within a fan on other repetitions, all of these occurrences will be reflected. Hence, an occurrence *O_v_* between 0 and 1 is assigned to each vector. The importance of this occurrence rate for the fiber direction is explained below in Section 2.5.

Broad fiber ODFs may be due to either fanning or curvature, and they are used to construct the PDF for the direction of propagation of tractography only when curve inference identifies them as fannings. In the case of curvature, the fiber ODF maximum is used as a discrete approximation of the curve, assuming the maximum represents the intermediate tangent to the curve segment in the voxel. This is illustrated in Figure 1. Box (b) will be the PDF for the direction of propagation of tractography in the case of curvature; box (c) will be the PDF in the case of fanning/merging. Box (d) illustrates how the streamlines will propagate, using FACT integration (19), in the cases of fanning (left) and merging (right). Box (e) illustrates how the streamlines will propagate in the case of curvature. The streamlines will enter and exit the voxel where the curve would have done.

### 2.4 Comparison of bootstrap variance to scan–rescan repeatability

Given an accurate description of the fiber geometry in a voxel, the remaining uncertainty in the fiber orientations is due to imaging noise. The residual bootstrap estimates this uncertainty. The ideal validation of the technique would be repeated scanning of the same subject, but obtaining enough repeated measurements to perform voxel-wise comparisons of the variability in the fiber orientation estimates is impractical. However, with O(10^5^) voxels in the brain, the degree to which the observed data match the bootstrap prediction for variability can be assessed on average for all voxels, despite the PDF for the fiber orientation being different at each voxel. If we have a bootstrap computation of the cones of uncertainty for a given confidence interval for the fiber ODF maxima, we expect it to predict the direction of the maxima of the fiber ODF for a subsequent registered MRI measurement. Hence, we expect the fiber ODF maxima computed from a subsequent scan to lie within the 95% confidence interval (see Figure 2) in 95% of the voxels in the brain.

Using one of the repeated diffusion-weighted scans, the PDF and confidence intervals for the fiber ODF maxima were obtained from bootstrapping. For a second, registered, MRI scan, the fiber ODF maxima obtained from spherical deconvolution were computed without bootstrapping. The number of voxels in which the fiber ODF maxima lay within the 68 and 95% confidence intervals obtained from the bootstrap analysis was counted. This was repeated for the other two coregistered datasets, and the whole process repeated four times for the four coregistered scans, meaning each scan was used for bootstrapping once, and the other three scans compared to it. The results were tabulated for fractional anisotropy (FA) ranges from 0.1 up. The threshold of FA >0.1 is expected to include many voxels with little or no white matter, but is often used for fiber tractography in pathways that go through, e.g., the thalamus, and other regions of partial volume averaging of fibers with other fibers or gray matter.

### 2.5 Tractography

The tractography algorithm used was an extended streamline tracking procedure implemented in a framework that considers the fiber orientation information obtained from the probabilistic curve inference labeling scheme. Tracking was initiated in a user-delineated seed ROI. Streamlines were propagated iteratively, with the direction of propagation chosen randomly from within the PDF for the direction of propagation of tractography described in Section 2.3. FACT (Fiber Assignment by Continuous Tracking) (19) integration was used. For each iteration, at each voxel reached, one fiber geometry was chosen at random from all geometries with non-zero occurrence. Next, for the case of crossing, the one fiber with mean ODF maximum direction closest to the incoming direction was chosen.

The direction of propagation was chosen at random from the PDF for the direction of propagation (see Figure 2) for the chosen fiber geometry, as described in Section 2.3. The *confidence value* for this vector tract segment was then given by the value of the PDF in this direction, scaled by a factor that reflects the confidence in this fiber direction’s existence. In the case of fanning, this scaling factor is the percent occurrence of the fanning geometry label. For all other cases, the scaling factor was the occurrence rate *O_v_* for the selected fiber direction. For example, if a single fiber is detected 60% of the time, and a second fiber that crosses it is detected 40% of the time, then the occurrence rate for the fiber direction detected all the time will be 100%, and for the fiber detected 40% of the time, the occurrence rate will be 40%. Hence, the confidence value will reflect our confidence that the fiber exists at all, independent of which specific geometry (in this example, single fiber or double crossing) it is part of. This is handled automatically in frequency of connection voxel-counting schemes, but needs to be handled explicitly here.

**Figure 3:**
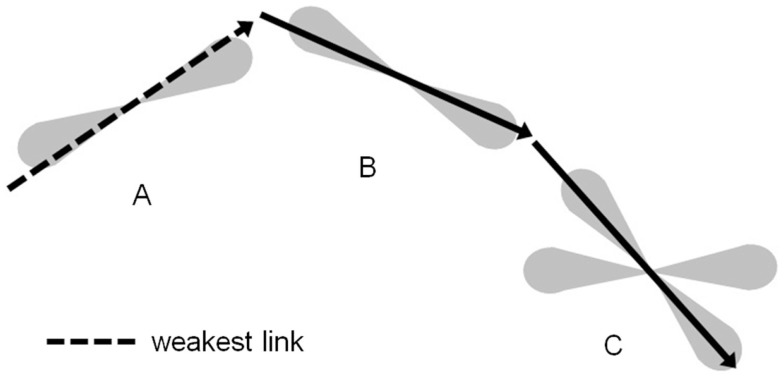
**The tractography process propagates streamlines iteratively with the direction of propagation chosen from within the PDF for the direction of fiber propagation**. The confidence in this tract segment generated is given by the scaled value of the PDF for the fiber orientation in the direction propagated. The confidence value for a streamline connecting voxel C to voxel A is given by the lowest confidence value for all segments along that streamline. Here, the dashed tract segment, which runs along the edge of the PDF, has the lowest confidence. The scalar connectivity index for connection of voxel C to reference voxel A will be given by the maximal confidence value of all streamlines that connect the two voxels.

**Figure 4:**
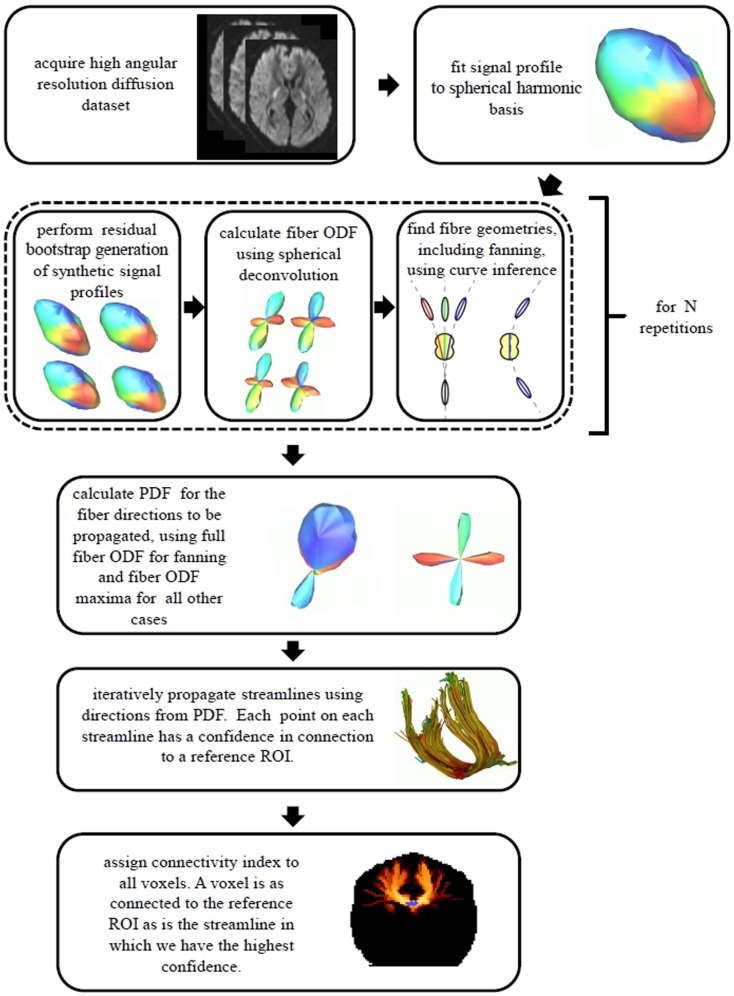
**A summary of the data processing pipeline for the combined curve inference labeling and bootstrap probabilistic tractography is shown**.

As the iterative tractography process evolved, confidence values were assigned to the streamlines using a *weakest link* approach (6, 22): the confidence in a given streamline is given by the lowest confidence value of all tract segments along the streamline. Before applying the minimum operation, the confidence values along the tract were blurred using a 1D Gaussian blurring kernel with a standard deviation of 2 mm, in order to avoid extreme sensitivity to isolated voxels. The connectivity index for each voxel in the imaging volume to a given reference ROI was then assigned: the connectivity index for a given voxel to the reference ROI is given by the highest confidence value over all the streamlines connecting voxels in the reference ROI to this voxel. Figure 3 illustrates this *weakest link* connectivity approach, and Figure 4 summarizes the processing for the combined curve inference labeling and residual bootstrap probabilistic pipeline.

Tractography experiments were run in different regions of the brain, using the *b* = 3000 s/mm^2^ data. Reference ROIs were drawn manually in the mid-sagittal corpus callosum, the cingulate bundle, the fornix, Broca’s area (the pars opercularis (area 44), and the pars triangularis (area 45) of the ventrolateral frontal cortex) (10), and the internal capsule. Tractography can be initiated in any seed ROI, but all experiments done here were seeded in the reference ROI. For all seed ROI voxels, the tracking was initiated on a 3 × 3 × 3 grid of start points in order to facilitate branching on the intervoxel scale. For each starting point, a large number of iterations of the probabilistic tractography were run in order to ensure convergence (1000 iterations were used in these experiments).

The tracking was stopped if the FA was less than 0.1, the mean diffusivity was greater than 1.0^−6^mm^2^/ms, or the turning angle from one voxel to the next was greater than a user-defined threshold. This curvature threshold was 80° for all experiments except the cingulum and fornix experiments, where it was 70°. For seed ROIs in the corpus callosum, fornix, and cingulum, tracts that erroneously turned down the cortical-spinal tract were excluded using exclusion masks. For the fornix and cingulum seeds, tracts that jumped onto the corticospinal tract or the corpus callosum were excluded. For the internal capsule tracking, tracts were seeded bilaterally in the internal capsule, and only tracts that connected to additional bilateral ROIs in the motor cortex were retained, excluding connections that passed through the mid-sagittal plane. For the seed ROI in Broca’s area, two experiments were run, one excluding commisural and projection fibers, and one with a second tract-delineating ROI in the homologous contralateral cortex. These stopping, exclusion, and inclusion criteria are user-defined and can be modified to reflect any prior knowledge the user has to the expected trajectory and curvature of the tract being reconstructed. In fact, tractography can be an interactive process in which criteria such as the curvature constraint are modified dynamically, but the criteria used must be well documented to aid interpretation of the results.

For comparison in several regions of major fiber tract systems where significant fanning was expected (the projections of the corpus collosum and cortical-spinal tract), the tracking was also done using the bootstrap probabilistic framework, but no curve inference, i.e., no treatment of fanning fibers. Connectivity index maps were stored for all experiments, with the connectivity index reflecting the confidence in connection of each voxel in the volume to the seed ROI.

## 3 Results

**Figure 5:**
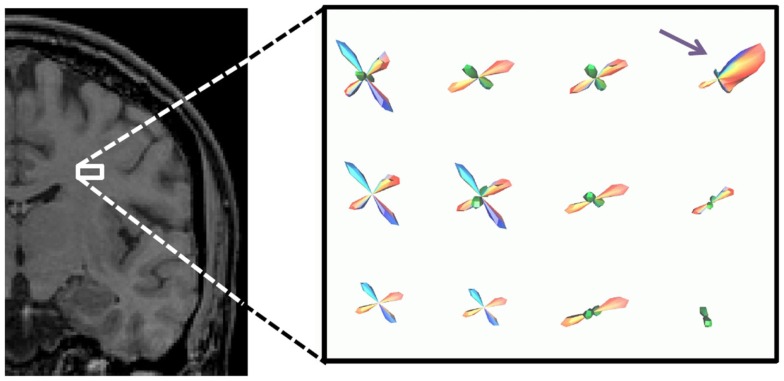
**PDF for the direction of propagation for tractography in a small ROI in the brain, shown by white box**. The fiber geometry with the highest occurrence is shown in each voxel. The ROI is at the decussation of the thalamo-cortical tract (blue), projections of the corpus callosum (red), and superior longitudinal fasciculus (green).

Figure 5 shows the computed PDF for the direction of propagation for tractography for the four different fiber configurations in a small ROI in the brain. The fiber geometry with the highest occurrence is shown in each voxel. The ROI is at the decussation of the thalamo-cortical tract (blue), projections of the corpus callosum (red), and superior longitudinal fasciculus (green); double and triple crossings of these fibers are seen, with the superior longitudinal fasciculus going through plane. Fibers of the corpus callosum fan to the right (purple arrow).

**Figure 6:**
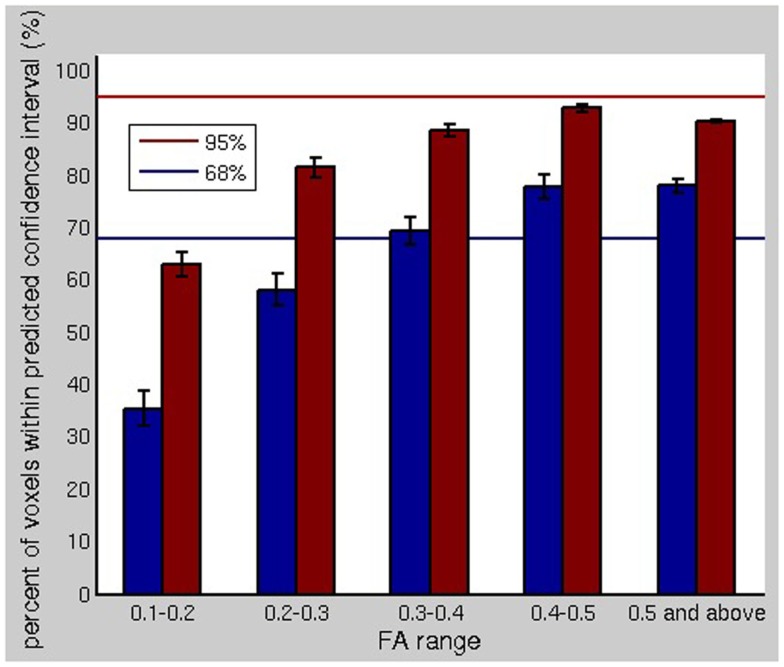
**The comparison of the bootstrap uncertainty profiles for fiber orientations is shown**. The blue and red bars show the percent of the voxels in the brain for which the fiber orientations, computed without bootstrapping, lay within the 68 and 95% bootstrap-predicted confidence intervals for the fiber orientation using a separate dataset. For major fiber tracts (i.e., FA >0.3), the correspondence between the observed variability in the fiber ODF maxima and the variability predicted by the bootstrap was very good.

Figure 6 shows the correspondence between observed and predicted confidence intervals for the fiber orientation estimates. We are comparing a single measurement (the fiber ODF maximum) to a distribution (the bootstrap PDF for the fiber ODF maximum) for each fiber at each voxel. The blue and red bars show the percent of the voxels in the brain for which the fiber orientations, computed without bootstrapping, lay within the 68 and 95% bootstrap-predicted confidence intervals for the fiber orientation. The blue and red lines indicate 68 and 95% on the *y*-axis, respectively. For major fiber tracts (i.e., FA >0.3), the correspondence between the observed variability in the fiber ODF maxima and the variability predicted by the bootstrap was very good, with the percentages matching closely. For low FA, the residual bootstrap underestimates the scan-rescan repeatability.

The underestimation of the scan-rescan repeatability could be due in part to the order eight SH expansion overfitting the noise at low FA values. It could also be attributed to slight misregistration between the acquired datasets, despite automated registration. The bootstrap predicts the variability due to noise, but cannot be expected to predict the variability due to subject positioning. Misregistration, including subvoxel misregistration, would be expected to affect the cores of major fiber pathways, which span multiple voxels, less than the edges (i.e., low FA), where there is significant partial volume averaging. The goal of the residual bootstrap processing is to predict the uncertainty in the fiber orientation(s) in order to propagate this uncertainty into fiber tractography results. Hence, subtle positioning changes could be expected to influence scan-rescan repeatability of tractography results more than predicted by residual bootstrap tractography, in tracking experiments using a low FA threshold. Otherwise, the residual bootstrap adequately predicts the variability due to noise.

**Figure 7:**
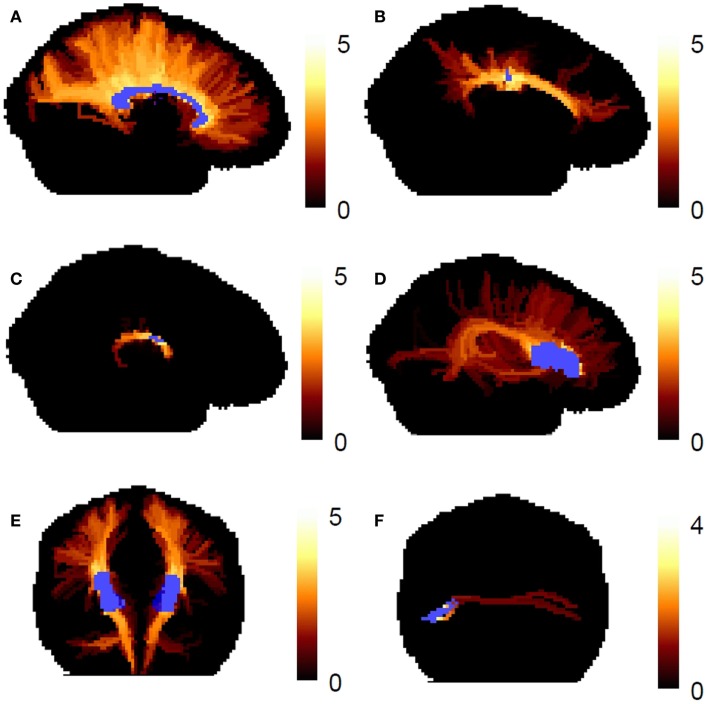
**The worst-case connectivity index map is shown in human fiber tract systems**. **(A)** Corpus callosum, **(B)** cingulate bundle, **(C)** fornix, **(D)** association connections of Broca’s area (left hemisphere), **(E)** corticospinal tracts, **(F)** transcallosal connections of Broca’s area. The connectivity index map is shown as a maximum intensity projection, and the reference ROIs are shown in blue.

Figure 7 shows the worst-case connectivity index in the fiber tract systems. The tracking follows the expected course in the major fiber bundles. For the association connections of Broca’s area, we see local cortico-cortical connections, the classic arcuate fasciculus, and connections to the temporal lobe through the extreme capsule, as have been seen in previous works (10). The expected transcallosal connections are also observed.

The highest connectivity values occur in the cores of major fiber tracts. For example, in the corpus callosum (Figure 7A), the highest connectivity values occur in the medial core of the corpus callosum, where the voxels contain large volume fractions of single fiber directions. This is expected because the reproducibility of the fiber ODF maximum is lower near the cortex than it is, e.g., in the middle of the corpus callosum. The connectivity index reflects our confidence in particular reconstructed streamlines, and this confidence will always drop in regions where the PDF for the fiber direction has more uncertainty. The lower confidence in the fiber direction for propagation near the cortex may occur because there is more partial volume averaging of fibers with other fiber populations and with gray matter and cerebral spinal fluid.

In Figure 7A, the reference ROI is the mid-sagittal corpus callosum, therefore the connectivity index values are high throughout the central region of the corpus callosum. When the reference ROI is cortical, as shown in Figure 7F, the streamlines have lower confidence segments early on their trajectory toward the contralateral cortex, and therefore, the connectivity index values are low throughout the entire course of the pathway, including the center of the corpus callosum (note color bar shows different scaling of connectivity index values compared to the rest of Figure 7).

**Figure 8:**
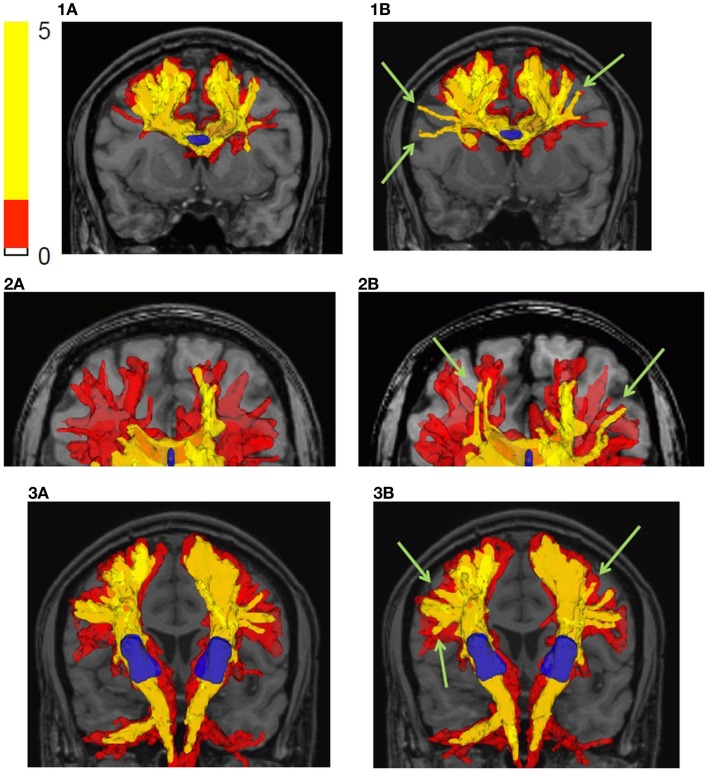
**Fiber tractography results in the human brain are shown**. (a) Bootstrap probabilistic framework, no curve inference labeling; (b) bootstrap probabilistic framework and curve inference labeling of fanning fibers. The results are shown by plotting isosurfaces of the connectivity index map superimposed on the T_1_ weighted anatomical image. The translucent red surface is that encompassing the lowest non-zero connectivity index values, and the yellow surface is a higher isosurface of the connectivity index map (shown in legend). Row (1) is the result using a seed ROI in the center of the corpus callosum, row (2) is the result starting in the genu of the corpus callosum, and row (3) is the result using an internal capsule seed ROI. The reference ROIs are shown in blue.

Figure 8 shows the tractography results using the bootstrap probabilistic framework but no curve inference labeling (left – a) and the results using the bootstrap probabilistic approach and curve inference labeling (right – b). Row (1) shows the results for a small multi-voxel seed ROI (shown in blue) in the center of the corpus callosum, row (2) shows the results in the genu of the corpus callosum, and row (3) shows the results for the seed ROI in the internal capsule. The reference ROIs are shown in blue, and two isosurfaces of the connectivity index map are shown, the red surface being that encompassing the lowest non-zero connectivity index values, and the yellow surface a higher isosurface of the connectivity index map.

The difference between incorporating fanning and not is subtle and manifests as a higher connectivity index for certain connections (see Figure 8, column b, green arrows); the extent of a given isosurface is greater in the case of fanning than without fanning. The explicit inclusion of fanning ameliorates the drop in fiber tract confidence near the cortex. This is expected because even when these voxels were reached in both tracking experiments, in the case of fanning, they were reached by propagating within the fan, where all confidence values are high. But without fanning information, the same voxels were reached by propagating near the edge of the PDF for the direction of a single fiber, where the confidence values are low. Note, however, that the streamlines have many opportunities to splay on the intervoxel scale with this approach, even when propagating only along the fiber ODF maximum instead of fanning on the intravoxel scale, because the dense subvoxel seeding on a grid facilitates splay. The differences between using fanning and not are more marked near the cortex and smaller in large fiber bundles.

The computation time for tractography depends highly on the size of the seed region, as well as the number of iterations and subvoxel seed density, but as run here, it takes on the order of 15 min for a seed ROI of roughly 100 voxels on a 2.13 GHz processor. The computation time for 100 repetitions of the probabilistic deconvolution without curve inference, i.e., without explicit identification of fanning, was approximately 30 min. With curve inference, the processing time is long – on the order of days for each bootstrap repetition on one processor. Hence, the total processing time depends on the number of processors used and the number of bootstrap repetitions.

## 4 Discussion

We have presented a residual bootstrap approach for tractography using a comprehensive description of possible subvoxel white matter fiber geometries. These geometries were straight, parallel fibers, curving fibers, crossing fibers, and fanning/merging fibers. Using the curve inference technique combined with spherical deconvolution, the iterative bootstrap process allowed us to define PDFs for the direction of fiber propagation for tractography, as seen in Figure 5. The bootstrap process was found to be an effective surrogate for multiple scanning sessions, as seen in Figure 6. This uncertainty was then propagated to the streamline tractography results (Figures 7 and 8).

The aim of this approach is to quantify uncertainty due to noise in the tracking results. There exist methods to reduce noise in the input data before running the processing described here. These approaches make assumptions such as similarity of different regions of the brain, and include linear minimum mean squared error and unbiased nonlocal means filters (7, 29). Such methods were not explored here, but could be useful in future investigations.

Quantifying uncertainty due to noise, and accurately modeling subvoxel fiber geometry, does not mean that we should have absolute faith in all results from the tractography, even connections to which we have assigned high confidence. Diffusion MRI data can, in the theoretical noise-free case, still support the existence of fiber connections that do not exist. When tracts pass close by each other, it is possible to jump from one tract system to another. This is a problem of spatial resolution for the most part. False positive results can be reduced by inputting user-defined priors. These include the exclusion masks and curvature constraints described here. User-input priors such as the curvature constraint can introduce bias in the tracking results (11), and the results change as these inputs change. Tractography is often a highly interactive process where the user inputs priors for curvature and excursion of the tracts. The inputs used here are not meant to be prescriptions, but rather to inform the viewer what priors were used so that the results can be interpreted with that knowledge.

We note that the fanning geometry incorporated in this analysis includes the case of branching. It is unlikely, at this resolution, that a sharp branch will occur on the subvoxel scale. Hence, if a fiber bundle is splitting into two bundles on a subvoxel scale, for example, this will present as a slow divergence of the two bundles, and cannot be differentiated from the slightly more uniform splay of fanning. This processing pipeline can easily be used to perform tractography using other complex descriptions of subvoxel geometries, such as bottlenecks, should the algorithms to describe these geometries be available. The curve inference algorithm used here treats only single fanning fibers, however, it is possible that fanning fibers might cross other fibers, and these other fibers might themselves fan. In fact, two fanning fiber systems could cross each other at 180°! Such cases, for instance, fibers fanning away from the cortex and fibers fanning toward the cortex, would require a different implementation.

The connectivity index used here, the weakest link approach, differs from other indices used in probabilistic tractography, such as the frequency of connection, which is obtained by counting the number of times a given voxel is reached by the iterative tracking process. The frequency of connection map can be non-monotonic in the case where a voxel is reached by two different pathways that merge at that voxel, i.e., a voxel can be more connected to the reference region than the voxels through which the connections occur. This feature may or may not be desired by the user. In the weakest link approach, these “hot spots” in the connectivity index map do not exist: a voxel cannot be more connected to the reference region than the voxels through which the connection passes, and a voxel is only as connected to the reference region as the most likely pathway between them.

In addition to the lack of “hot spots,” the confidence values obtained with the weakest link approach are independent of the size and shape of the tract-delineating ROIs, which is not the case with the frequency of connection approach. In the frequency of connection approach, extending the seed ROI parallel to the direction of the fiber pathway will result in a pileup of connections, hence higher connectivity indices. In contrast, the weakest link connectivity index reflects the integrity of the strongest voxel to voxel connection, and we have only as much confidence in this pathway as in the segment thereof in which we have the least confidence. The weakest link approach is also less corrupted by distance effects, which give a strong bias to shorter connections in the frequency of connection approach (20).

In summary, this pipeline consists of many steps that are combined to produce maps of our confidence that the diffusion MRI data support the existence of a connection between any two regions of the brain. MRI acquisition designed for high angular resolution, spherical deconvolution, curve inference, and a bootstrap probabilistic framework are combined to produce PDFs for the direction of propagation of tractography. These PDFs are then used, with an intuitive worst-case connectivity index, to create maps of our confidence in white matter connectivity. The pipeline is useful with and without the use of curve inference to identify fanning, but explicit identification of fanning increases the inferred confidence in connections near the cortex. If time does not permit, the pipeline can be run without the curve inference step as shown in Figure 8 (column a), with the benefit that the processing time is much shorter and suitable for fast analysis and large cohorts. The pipeline is a way to perform quality control on the uncertainty due to noise and visualize how this propagates into the tractography result.

These tractography results demonstrate the ability of the proposed probabilistic tractography pipeline to describe fiber pathways that pass through regions of complex subvoxel geometries. Accurately describing the fiber geometries and propagating the uncertainties in these geometries through to the final tractography map facilitates interpretation of this map. Coupled with this more complete description of possible fiber geometries, the weakest link connectivity index provides a robust and logical approach for the description of white matter connectivity *in vivo*. As opposed to existing tractography approaches that do not identify fanning (3), or essentially assume splay of fibers in both directions when the fiber ODF is broad (28), this approach can increase both sensitivity and specificity of the tractography results.

## Author Contributions

Jennifer S. W. Campbell and Parya MomayyezSiahkal were responsible for the experimental design, coding, most experiments, and manuscript writing. Peter Savajiev designed and wrote the curve inference algorithm and provided feedback on this application. Ilana R. Leppert performed tractography experiments and provided feedback. Kaleem Siddiqi and G. Bruce Pike provided supervision for the project. All authors approved the manuscript.

## Conflict of Interest Statement

The authors declare that the research was conducted in the absence of any commercial or financial relationships that could be construed as a potential conflict of interest.

## Footnotes

^1^The code is currently available upon request from the authors.
